# Can PIRCHE-II Matching Outmatch Traditional HLA Matching?

**DOI:** 10.3389/fimmu.2021.631246

**Published:** 2021-02-26

**Authors:** Christian Unterrainer, Bernd Döhler, Matthias Niemann, Nils Lachmann, Caner Süsal

**Affiliations:** ^1^Institute of Immunology, Heidelberg University Hospital, Heidelberg, Germany; ^2^PIRCHE AG, Berlin, Germany; ^3^Institute for Transfusion Medicine, HLA-Laboratory, Charité-Universitätsmedizin Berlin, Berlin, Germany

**Keywords:** epitope matching, HLA matching, PIRCHE-II, kidney allocation, presensitization, Collaborative Transplant Study

## Abstract

We analyzed in a cohort of 68,606 first deceased donor kidney transplantations reported to the Collaborative Transplant Study whether an epitope-based matching of donor-recipient pairs using the Predicted Indirectly ReCognizable HLA Epitopes algorithm (PIRCHE-II) is superior to currently applied HLA antigen matching. PIRCHE-II scores were calculated based on split antigen HLA-A, -B, -DRB1 typing and adjusted to the 0–6 range of HLA mismatches. PIRCHE-II scores correlated strongly with the number of HLA mismatches (Spearman ρ = 0.65, *P* < 0.001). In multivariable analyses both parameters were found to be significant predictors of 5-year death-censored graft loss with high prognostic power [hazard ratio (HR) per adjusted PIRCHE-II score = 1.102, per HLA mismatch = 1.095; *z*-value PIRCHE-II: 9.8, HLA: 11.2; *P* < 0.001 for both]. When PIRCHE-II scores and HLA mismatches were analyzed simultaneously, their predictive power decreased but remained significant (PIRCHE-II: *P* = 0.002; HLA: *P* < 0.001). Influence of PIRCHE-II was especially strong in presensitized and influence of HLA mismatches in non-sensitized recipients. If the level of HLA-incompatibility was low (0–3 mismatches), PIRCHE-II scores showed a low impact on graft survival (HR = 1.031) and PIRCHE-II matching did not have additional significant benefit (*P* = 0.10). However, if the level of HLA-incompatibility was high (4–6 mismatches), PIRCHE-II improved the positive impact of matching compared to applying the traditional HLA matching alone (HR = 1.097, *P* = 0.005). Our results suggest that the PIRCHE-II score is useful and can be included into kidney allocation algorithms in addition to HLA matching; however, at the resolution level of HLA typing that is currently used for allocation it cannot fully replace traditional HLA matching.

## Introduction

Human leukocyte antigen (HLA) matching continues to be part of the major kidney allocation systems. Under insufficient immunosuppression, HLA mismatches can lead to activation of alloreactive T-helper cells that support cytotoxic T cells, B cells and antibody producing plasma cells that are capable of harming the transplant. Pre-transplant presence and post-transplant development of alloantibodies against highly polymorphic HLA antigens have been shown to play a major role in rejection of kidney allografts and recent data support that, even under the currently applied potent immunosuppression, matching for HLA antigens is beneficial, also in recipients of kidneys from elderly donors, and significantly reduces important adverse outcomes, such as graft loss, mortality, rejection episodes, and development of non-Hodgkin lymphoma (1–6).

In the Eurotransplant region, recipients and donors are matched based on alleles from three different HLA loci, namely HLA-A, -B, and -DRB1. However, the immune system does not recognize the whole foreign HLA allele molecule but small sequences of it, called epitopes. Moreover, the same antigenic epitope can be present on different alleles, which further complicates the evaluation of compatibility. To improve the precision of matching between donor and recipient and the knowledge on HLA incompatibilities that are relevant for rejection of kidney allografts, several theoretical and experiment-based algorithms have been developed (7–11). The Predicted Indirectly ReCognizable HLA Epitopes (PIRCHE-II) algorithm is one of these promising theoretical approaches and matching based on this algorithm was shown to have potential to improve graft survival (12). It allows calculation of an epitope load score based on donor HLA epitopes that can be presented by HLA-DRB1 molecules of the recipient, but are not found in the recipient's own HLA-A, -B, -C, -DRB1 and -DQB1 alleles, thus modeling the indirect pathway of allorecognition by CD4+ T cells.

In a single center analysis of 2,787 consecutive kidney transplantations performed during 1995–2015, Lachmann et al. reported in 2017 that the PIRCHE-II score, independently from the currently used HLA antigen matching, is a strong predictor of *de novo* development of donor-specific HLA antibodies (dnDSA), whereas the prediction of allograft survival by PIRCHE-II score was, although statistically significant, rather moderate, probably also due to the limited number of patients in the decisive groups (13). Analyzing a similarly sized multi-center cohort of 2,918 kidney transplantations transplanted during 1995–2005 in the Netherlands, Geneugelijk et al. reported in 2018 that a high PIRCHE-II score is associated with a clearly higher risk of graft failure, while in their univariate model the predictive power of PIRCHE-II was stronger than that of HLA mismatches (12). These promising results stimulated us to analyze in a large collective of more than 65,000 kidney transplantations whether the PIRCHE-II score matching is superior to the traditional HLA antigen matching and has even the potential to replace it. The PIRCHE-II score has the capacity to be more precise than the traditional HLA antigen matching, alone due to the fact that it comes with a higher range of values (0–211 in our study) compared to the possible seven mismatch categories (0–6) in HLA-A, -B, -DRB1 matching. However, also the PIRCHE-II score is calculated based on HLA alleles and the comparative evaluation of two similar parameters that aim to assess the impact of the same phenomenon, namely the immunological difference between donor and recipient, is difficult because no statistical test exists that allows comparison of hazard ratios (HR). This applies irrespective of whether the HRs are calculated in different Cox regression models or stem from the same model. One solution for a reliable comparison is to adjust the PIRCHE-II scores to the same 0–6 range as HLA A+B+DRB1 mismatches and to analyze the impact of one parameter in subcategories of the other in different multivariable models of Cox regression. Such a cross-analysis was possible using the large database of the Collaborative Transplant Study (CTS) which contains sufficient number of patients in each subcategory.

## Materials and Methods

First European kidney-only transplantations from deceased donors reported to CTS (www.ctstransplant.org) and performed during 1990–2016 with available information on recipient and donor age, cold ischemia time and one-field split donor and recipient HLA-A, -B, -DRB1 typing were analyzed. The PIRCHE-II scores for HLA-A, -B and -DRB1 loci were calculated blinded by PIRCHE AG (Berlin, Germany) and sent back to CTS for further analysis. For solid organ transplantation, the PIRCHE-II score describes the number of unique 15-mer peptides of HLA proteins that are encoded in exon 2 to 5 of donor HLA genes, are not present in the peptide repertoire of the recipient's self-HLA proteins and are likely to be presented by HLA-DRB1 proteins of the recipient. Identical 9-mer cores of different 15-mer allele sequences are counted only once per presented protein (14).

For the calculation of PIRCHE-II scores the National Marrow Donor Program (NMDP) EUR haplotypes from 2007 were used. The standard deviations reflect the accuracy with which the PIRCHE-II score was calculated as described by Geneugelijk et al. (15). The standard deviation is based on the possible two-field molecular HLA typings and haplotypes that can be deducted from the split HLA typings of donor and recipient weighted by their frequency in the NMDP database.

The magnitude of the HLA and PIRCHE-II score effects are not comparable using the HRs because the two variables are on different scales; HLA A+B+DRB1 mismatches range from 0 to 6 whereas PIRCHE-II scores ranged in our cohort from 0 to 211. In order to achieve comparable hazard ratios in the same 0–6 scale and to account at the same time for the previously published logarithmic feature of the PIRCHE-II effect (13), PIRCHE-II score for patient ‘i' was adjusted using the formula

$$\begin{array}{l} Pirche_{adj,i}=Ln\left(Pirche_{i}+1\right)\;*\;\frac{6}{\max\left(Ln\left(Pirche+1\right)\right)} \end{array}$$

Pirche_adj, i_ represents the adjusted PIRCHE-II score of patient “i” and Pirche the vector of PIRCHE-II scores of all 68,606 patients. The adjusted PIRCHE-II scores were categorized to obtain similarly sized PIRCHE-II score and HLA A+B+DRB1 mismatch groups. In a subgroup analysis, the seven PIRCHE-II categories 0–1 (4,407 patients), 2–12 (5,223), 13–25 (13,643), 26–43 (19,996), 44–68 (16,424), 69–102 (7,511), and >102 (1,402) were compared with the seven HLA mismatch categories 0 (4,469), 1 (5,297), 2 (13,282), 3 (20,096), 4 (16,171), 5 (7,401), and 6 (1,890).

For testing of categorized variables chi-squared-test and for comparison of continuous variables Kruskall-Wallis-test was used. As statistical software SAS, R and the survival package in R were used (16–18). To analyze the impact of PIRCHE-II scores and HLA A+B+DRB1 mismatches on death-censored graft survival, multivariable Cox regression analyses were performed considering the following confounders: recipient and donor age, recipient and donor sex, time on dialysis, disease leading to transplantation, latest panel reactive antibodies, donor hypertension or other reason for marginal donor, induction therapy, initial immunosuppression, transplant year, and cold ischemia time. Country-based stratification was applied. Missing data in confounding factors, such as panel reactive antibodies, time on dialysis and immunosuppression, were considered as a separate category. Depending on the analysis performed, either the adjusted PIRCHE-II scores, HLA mismatches or both variables together were added to this Cox model. In total, three models were applied. In the first model all mentioned basic confounders and HLA mismatches, in the second model all basic confounders and PIRCHE-II score and in the third model (full) all basic confounders and HLA mismatches as well as PIRCHE-II score were included. In order to compare their individual overall influence, the impact of PIRCHE-II scores and HLA mismatches were first analyzed separately. However, by the nature of their origin, PIRCHE-II scores and HLA A+B+DRB1 mismatches are not statistically independent. There was no patient with 6 HLA A+B+DRB1 mismatches who had a PIRCHE-II score of 0 and conversely, patients with a high PIRCHE-II score of >100 had in none of the cases 0 HLA A+B+DRB1 mismatches. Therefore, we applied also a Cox regression model in which both parameters were analyzed as confounding factors simultaneously. In addition, the effect of one factor was analyzed in categories of the other variable.

For comparison of the prediction capacity of different Cox models, the Akaike's information criterion (AIC) is given in Table 2. ROC analysis of 5-year graft survival for the full model was performed using the R package pROC (19).

## Results

**Figure 1:**
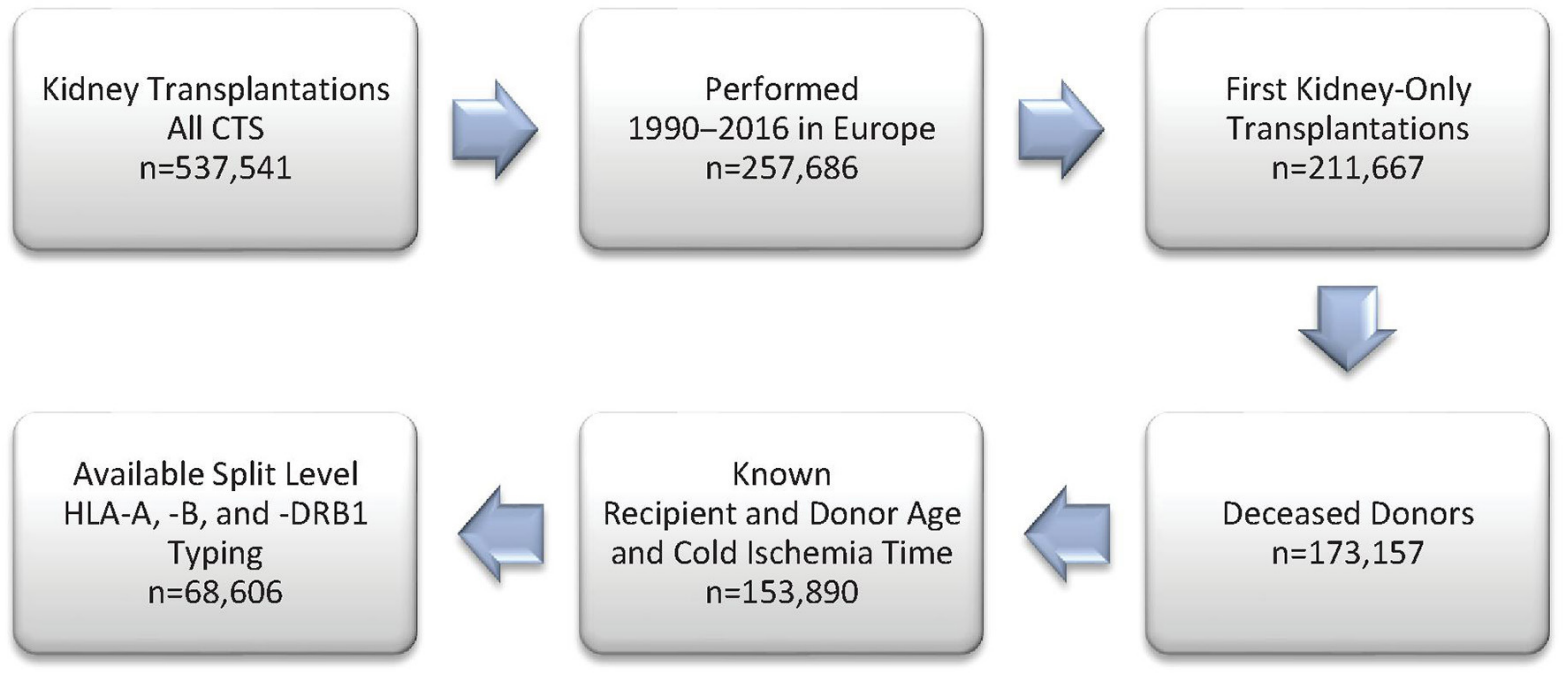
Study flow chart of included patients from the Collaborative Transplant Study (CTS) database.

**Figure 2:**
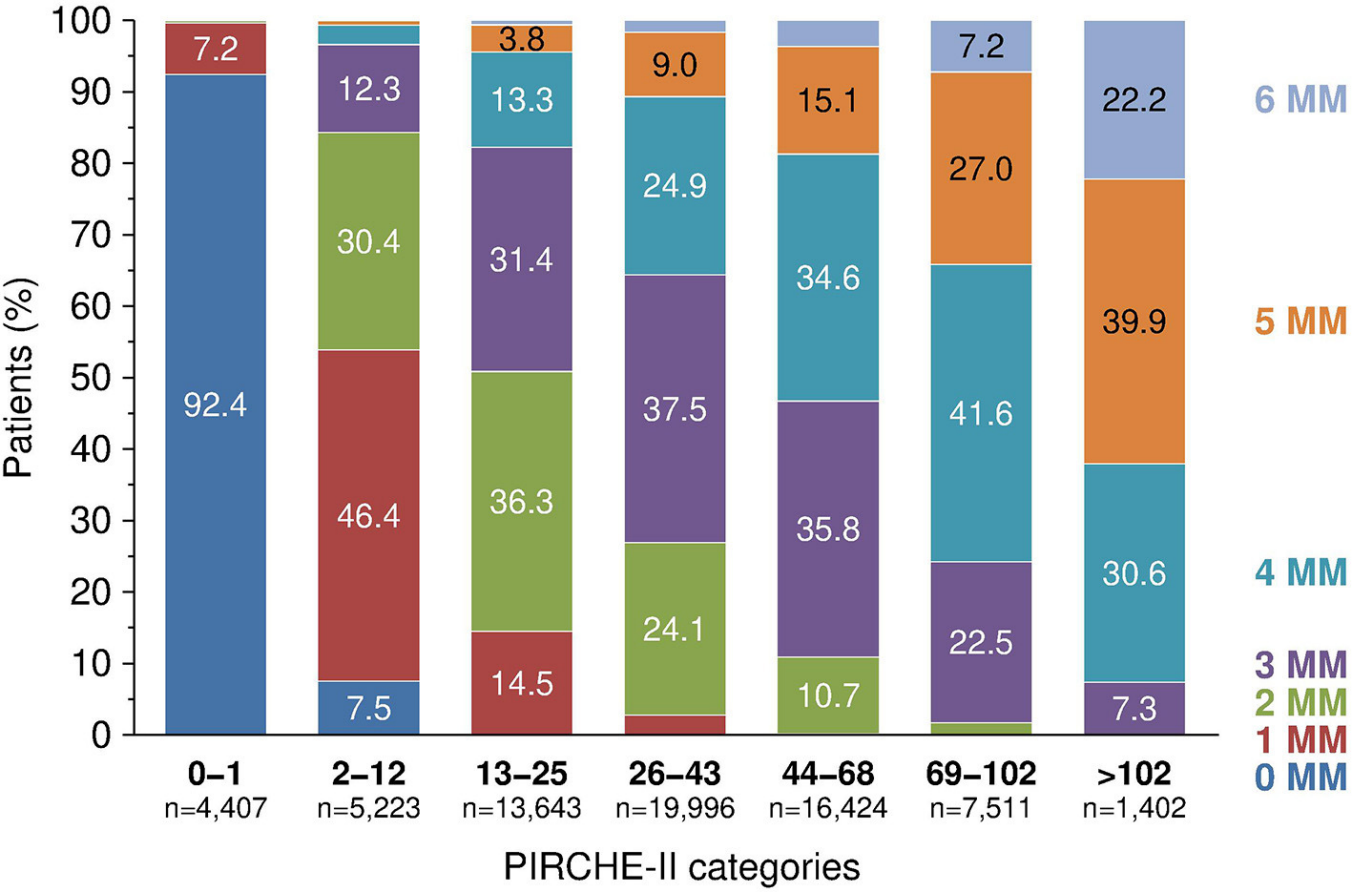
The relationship between PIRCHE-II score categories and fraction of patients with different number of HLA A+B+DRB1 mismatches (MM), *P* < 0.001.

Figure 1 shows the selection process for this analysis in a flowchart. The demographics of 68,606 deceased donor kidney transplantations with a mean follow-up of 6.9 years performed at 236 European CTS centers in 24 countries and categorized according to adjusted PIRCHE-II scores are shown in Table 1 and in more detail in Supplementary Table 1. As expected, PIRCHE-II score correlated positively with the number of HLA A+B+DRB1 mismatches (Spearman's rank correlation coefficient ρ = 0.65, *P* < 0.001; Figure 2). As many as 92.6% of patients with a very low PIRCHE-II score of 0–1 had 0 HLA A+B+DRB1 mismatches and many patients (62.1%) with a high PIRCHE score of >102 had 5 or 6 HLA mismatches. None of the patients with 0 HLA mismatches showed a high PIRCHE-II score of ≥20 and conversely, only 0.7% of patients with 5–6 HLA mismatches had a PIRCHE-II score below 13. Overall, with increasing HLA mismatches of 0, 1, 2, 3, 4, 5, and 6 also the variance of PIRCHE-II scores increased with standard deviations of 1.1, 9.2, 14.2, 19.2, 23.3, 27.3, and 32.2, respectively. In contrast, the standard deviation of HLA mismatches remained with around 1 relatively constant in all seven PIRCHE-II score categories, also due to the limited number of possible HLA mismatch outcomes (0 to 6) compared to the larger pool of possible PIRCHE-II scores (0 to 211 in our study).

**Table 1:** Baseline characteristics of 68,606 first deceased donor kidney transplantations in categories of the PIRCHE-II score.

**Confounder**	**PIRCHE-II score**			**Unknown**	***P***
	**0–12 *n* = 9,630**	**13–68 *n* = 50,063**	**69–211 *n* = 8,913**		
**Year of transplantation**				0	<0.001
1990–1996	1,764 (18.3%)	6,776 (13.5%)	907 (10.2%)		
1997–2003	3,207 (33.3%)	14,373 (28.7%)	2,307 (25.9%)		
2004–2010	2,829 (29.4%)	15,280 (30.5%)	2,830 (31.8%)		
2011–2016	1,830 (19.0%)	13,634 (27.2%)	2,869 (32.2%)		
**Recipient sex**				0	<0.001
Male	5,848 (60.7%)	31,506 (62.9%)	5,779 (64.8%)		
Female	3,782 (39.3%)	18,544 (37.0%)	3,134 (35.2%)		
**Recipient age (years)**					<0.001
Mean (SD)	48.0 (14.2)	49.0 (15.2)	51.3 (15.3)		
**Donor sex**				81	0.013
Male	5,356 (55.6%)	28,551 (57.0%)	5,044 (56.6%)		
Female	4,257 (44.2%)	21,463 (42.9%)	3,854 (43.2%)		
**Donor age (years)**					<0.001
Mean (SD)	45.2 (15.7)	47.4 (17.5)	49.6 (18.6)		
**HLA A+B+DRB1 mismatches**				0	<0.001
0–1	7,203 (74.8%)	2,563 (5.1%)	0 (0.0%)		
2–4	2,392 (24.8%)	41,682 (83.3%)	5,475 (61.4%)		
5–6	35 (0.4%)	5,818 (11.6%)	3,438 (38.6%)		
**Cold ischemia time (hours)**				0	<0.001
Mean (SD)	17.9 (7.03)	17.1 (7.03)	16.3 (6.80)		
**Panel reactive antibodies (%)**				16,064	0.052
=0	6,184 (64.2%)	31,792 (63.5%)	5,911 (66.3%)		
>0	1,134 (11.8%)	6,337 (12.7%)	1,184 (13.3%)		
**Time on dialysis (months)**				14,803	0.12
No dialysis	181 (1.9%)	1,063 (2.1%)	199 (2.2%)		
Mean (SD)	43.0 (33.7)	45.4 (38.0)	45.3 (38.0)		
**Initial immunosuppression**				4,033	<0.001
Tac + MPA	2,597 (27.0%)	16,635 (33.2%)	3,306 (37.1%)		
CsA + MPA	2,473 (25.7%)	11,310 (22.6%)	2,088 (23.4%)		
Other	4,190 (43.5%)	19,080 (38.1%)	2,894 (32.5%)		
**Initial induction therapy**				4,033	<0.001
ATG	751 (7.8%)	5,032 (10.1%)	1,067 (12.0%)		
IL-2RA	1,731 (18.0%)	12,659 (25.3%)	2,706 (30.4%)		
No induction	6,596 (68.5%)	28,189 (56.3%)	4,261 (47.8%)		
Other	182 (1.9%)	1,145 (2.3%)	254 (2.8%)		

*For categorized variables P-values of chi-squared-test and for continuous variables P-Values of Kruskal-Wallis-test are shown*.

*SD, standard deviation; Tac, tacrolimus; MPA, mycophenolic acid; CsA, cyclosporine A; ATG, anti-thymocyte globulin; IL-2RA, interleukin-2 receptor antagonist*.

To account for the several confounders of death-censored graft survival, multivariable Cox regression analyses were performed. We analyzed first the predictive power of PIRCHE-II scores and HLA mismatches on death-censored kidney graft survival separately by considering only one of these two parameters with all other confounders. The complete model with PIRCHE-II scores and HLA in addition to the basic parameters showed an AUC of 0.67 for 5-year graft survival (Supplementary Figure 1). This is in line with the results of a UNOS analysis in which an AUC of 0.66 was found for 3-year graft survival using a multivariable Cox regression model with UNOS parameters (20). As shown in Table 2, both parameters were found to be significant predictors of 5-year death-censored graft loss with high prognostic power. The calculated HR per adjusted PIRCHE-II score was with 1.102 slightly higher than the 1.095 HR calculated per HLA A+B+DRB1 mismatch. In contrast, however, the Wald statistic z-value (Cox regression coefficient divided by its standard error) as a measure of the certainty of the observed effect was higher for HLA mismatches than for adjusted PIRCHE-II scores (11.2 vs. 9.8; *P* < 0.001 for both). When the impact of adjusted PIRCHE-II score and HLA mismatches on outcome was analyzed simultaneously, both parameters remained statistically significant (*P* = 0.002 and < 0.001, respectively), whereas the magnitude of their influence, as expected, decreased; the HR for graft loss per adjusted PIRCHE-II score dropped from 1.102 to 1.043 and the z-value from 9.8 to 3.0. A similar but less pronounced decrease was observed for HLA mismatches; the HR per mismatch dropped from 1.095 to 1.069 and the z-value from 11.2 to 5.8 (Table 2).

**Table 2:** Impact of matching for PIRCHE-II score and HLA A+B+DRB1 on 5-year death-censored graft survival of kidney transplant recipients in different multivariable Cox regression models.

**Model**	**HR**	**95% CI**	**Z**	***P***
**Without HLA A+B+DRB1 mismatches (AIC** **=** **150,629)**				
Per adjusted PIRCHE-II score	1.102	1.081–1.123	9.8	<0.001
**Without adjusted PIRCHE-II score (AIC** **=** **150,605)**				
Per HLA A+B+DRB1 mismatch	1.095	1.078–1.113	11.2	<0.001
**Simultaneously with both parameters (AIC** **=** **150,598)**				
Per adjusted PIRCHE-II score	1.043	1.015–1.071	3.0	0.002
Per HLA A+B+DRB1 mismatch	1.069	1.045–1.093	5.8	<0.001

*HR, hazard ratio; CI, confidence interval; Z, Wald statistic value; AIC, Akaike Information Criterion*.

**Figure 3:**
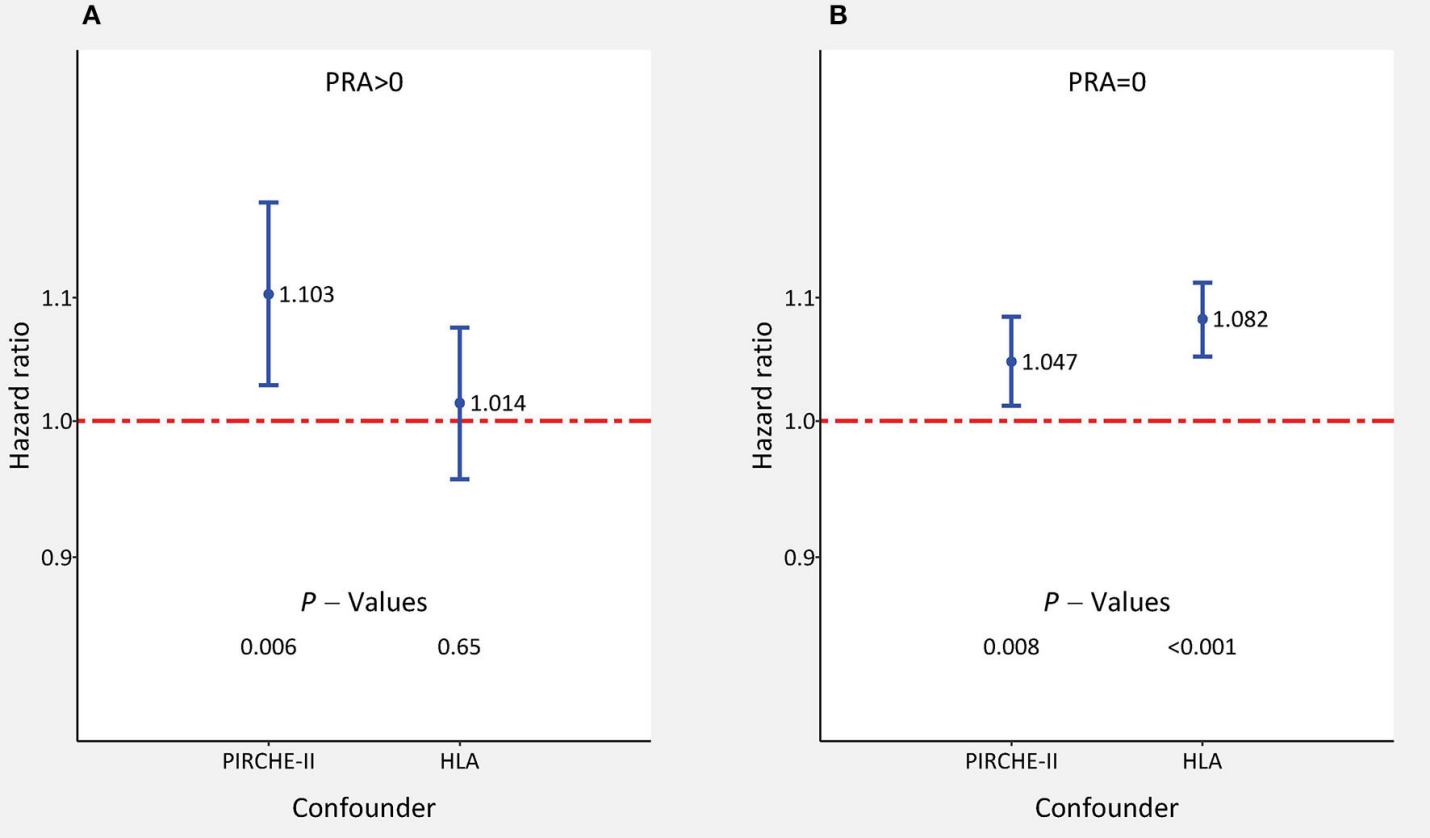
The impact of adjusted PIRCHE-II scores and HLA mismatches on 5-year death-censored graft survival in subgroups of kidney transplant recipients **(A)** with and **(B)** without panel reactive lymphocytotoxic antibodies (PRA). Hazard ratios ±95% confidence interval of multivariable Cox regression with simultaneous consideration of all confounders are shown.

We analyzed the influence of both parameters further simultaneously, and for comparison also separately, in different subgroups of patients with impaired outcome. It must be noted that when both parameters are considered simultaneously, the absence of statistical significance for one parameter does not necessarily mean that its impact in this subgroup is missing because both parameters are measures of HLA-compatibility and the influence can be hidden in the other parameter which shows a significant influence. When analyzed separately, both parameters had a high impact on outcome in most of the analyzed subgroups (Supplementary Table 2). When considered simultaneously with HLA mismatches, PIRCHE-II score had an especially strong influence on 5-year death-censored graft survival in patients with an assumed higher alloreactivity, such as presensitized patients with lymphocytotoxic panel reactive antibodies (PRA >0%). The influence of PIRCHE-II score on outcome was more than twice as high in presensitized compared to non-sensitized patients (HR = 1.103 vs. 1.047; *P* = 0.006 and 0.008, respectively). The impact of HLA mismatches on outcome was, in contrast, more pronounced in non-sensitized compared to presensitized patients (HR = 1.082 and 1.014; *P* < 0.001 and 0.65, respectively; Figure 3). Compared to the 1.041 HR value in the adult cohort, the 1.092 HR value for the influence of PIRCHE-II score was also more than twice as high in the pediatric cohort; however, most probably due to the low number of cases, the result did not reach statistical significance (*P* = 0.004 and 0.18, respectively). In contrast, the impact of HLA mismatches on outcome was stronger in adult than pediatric recipients (1.066 and 1.028; *P* < 0.001 and 0.62, respectively). Prolonged ischemia time >18 h or older donor age ≥60 years did not appear to influence the impact of PIRCHE-II scores or HLA mismatches greatly (Supplementary Table 2).

**Figure 4:**
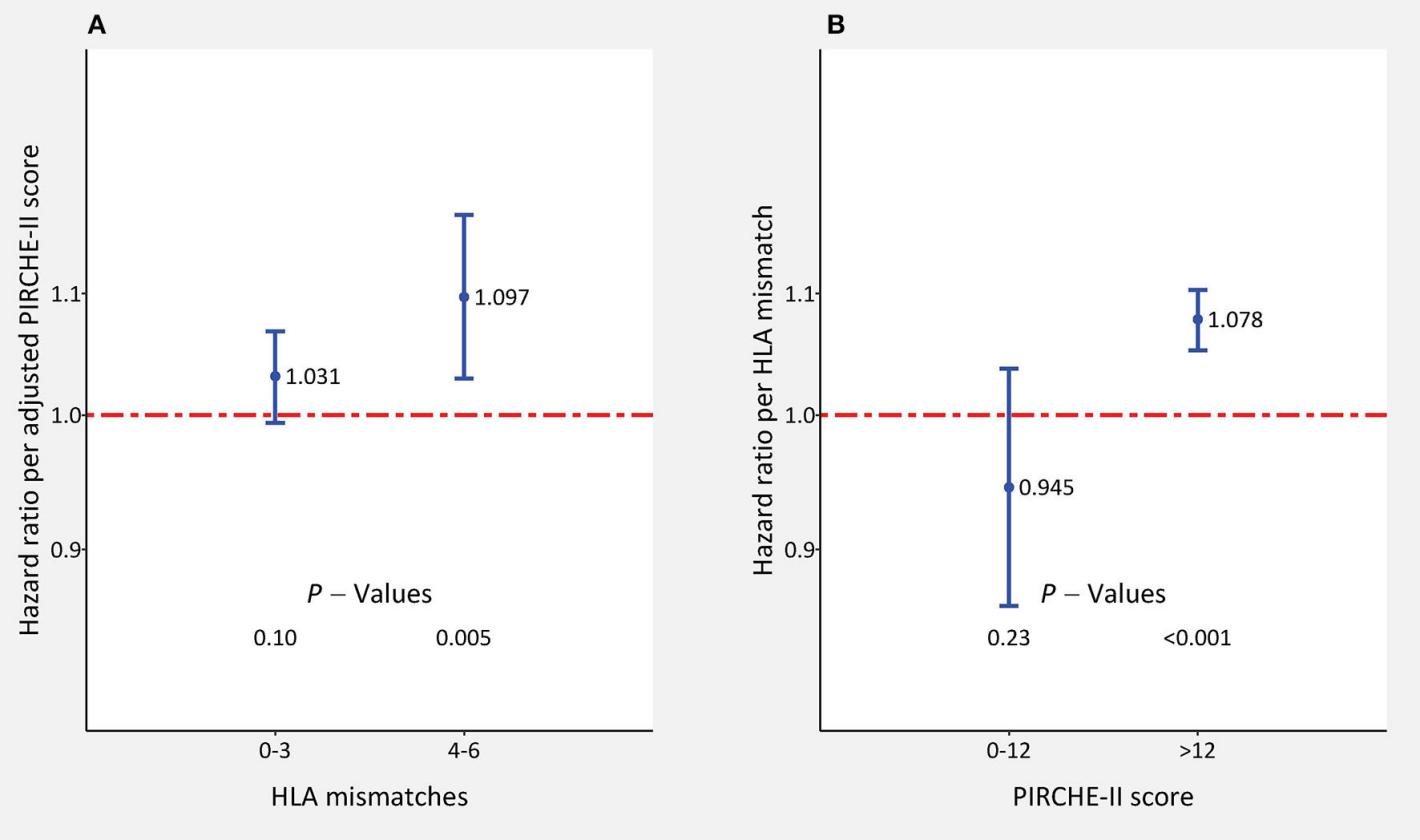
The impact of adjusted PIRCHE-II scores and HLA mismatches on 5-year death-censored graft survival in kidney transplant recipients with low (0–3; *n* = 43,144) and high number (4–6; *n* = 25,462) of **(A)** HLA mismatches or **(B)** 0–12 (*n* = 9,630) and >12 (*n* = 58,976) PIRCHE-II scores. Hazard ratios ±95% confidence interval per score point are shown. HLA mismatches and adjusted PIRCHE-II scores were analyzed simultaneously in the multivariable Cox regression model.

We studied also in detail the impact of adjusted PIRCHE-II scores for each HLA mismatch value and of HLA mismatches for each PIRCHE-II category (Table 3). The HLA-mismatch value was a significant predictor of graft survival in all PIRCHE-II score categories above 12, whereas the PIRCHE-II score was only significant in the 5 HLA-mismatch category but reached above 3 mismatches HRs of 1.063–1.074 which were not very much different from the HRs of significant HLA-mismatch results (1.055–1.236). As illustrated in Figure 4A, PIRCHE-II matching did not show an additional significant benefit for death-censored graft survival if the level of HLA-incompatibility was with 0–3 mismatches low (HR = 1.031; *P* = 0.10). However, if the level of HLA-incompatibility was high (4–6 mismatches), PIRCHE-II improved the positive impact of matching compared to applying the traditional HLA matching alone (HR = 1.097, *P* = 0.005). Similarly, as shown in Figure 4B, also the HLA mismatches did not influence the outcome in low PIRCHE-II categories 0–12 significantly (HR = 0.945; *P* = 0.23) but had a significant effect on death-censored graft survival in PIRCHE-II categories >12 (HR = 1.078; *P* < 0.001). These findings altogether indicated that both parameters deliver additional complementary information regarding outcome in the higher score categories of the other parameter, but the additional benefit remains uncertain in the lower score categories.

**Table 3:** Impact of adjusted PIRCHE-II scores and HLA mismatches on 5-year death-censored graft survival in kidney transplant recipients with different number of HLA mismatches or PIRCHE-II scores.

**Subgroup**	**HR**	**95% CI**	***P***
**HR per adjusted PIRCHE-II score**			
0 HLA mismatches	0.907	0.741–1.108	0.34
1 HLA mismatch	1.033	0.945–1.129	0.47
2 HLA mismatches	1.016	0.940–1.099	0.69
3 HLA mismatches	1.029	0.960–1.104	0.42
4 HLA mismatches	1.063	0.979–1.154	0.15
5 HLA mismatches	1.179	1.048–1.325	0.006
6 HLA mismatches	1.074	0.858–1.344	0.54
**HR per HLA A+B+DRB1 mismatch**			
0–1 PIRCHE-II score	1.123	0.826–1.528	0.46
2–12 PIRCHE-II score	0.913	0.826–1.008	0.073
13–25 PIRCHE-II score	1.070	1.021–1.122	0.005
26–43 PIRCHE-II score	1.055	1.014–1.098	0.009
44–68 PIRCHE-II score	1.095	1.046–1.146	<0.001
69–102 PIRCHE-II score	1.110	1.032–1.194	0.005
>102 PIRCHE-II score	1.236	1.048–1.458	0.012

*The third Cox regression model as described in the Methods section was used. Hazard ratios (HR) and 95% confidence intervals (CI) per adjusted PIRCHE-II score or per HLA A+B+DRB1 mismatch are shown*.

## Discussion

In order to be precise, most of the epitope matching algorithms, including PIRCHE-II, require two-field molecular HLA typing. However, Geneugelijk et al. reported that in a Caucasian population, even at split HLA antigen typing level, the calculation of the PIRCHE-II score is accurate if performed using multiple imputation with haplotype frequencies (15). This is important because e.g., the currently used allocation algorithm by Eurotransplant is also based on HLA typing at serological broad (HLA-A and HLA-B) and split antigen (HLA-DRB1) level and not at the level of more precise two-field molecular typing. Therefore, we found it of interest to analyze whether the PIRCHE-II score is applicable to the typing environment of the currently used matching algorithms for kidney allocation, with the exception that in our study also the HLA-A and HLA-B loci were analyzed at a higher, namely at the split instead of broad antigen level. Despite the availability of haplotype frequency datasets of non-Caucasian haplotypes, we limited our analysis to European transplantations as the aforementioned multiple imputation has not been validated in other populations yet. Therefore, our results may not be representative for other ethnic groups and accuracy is expected to decrease in more diverse patient collectives or when suitable haplotype frequency tables are unavailable.

In the present study, we subjected the PIRCHE-II score that was based on HLA-A, -B, and -DRB1 typing to a reality check in a large cohort of more than 65,000 kidney transplantations performed during 1990–2016. We sent our pseudonymized typing data to PIRCHE AG and received back a list of PIRCHE-II scores and their individual standard deviations derived from multiple imputation. When the adjusted PIRCHE-II score and HLA A+B+DRB1 mismatches were analyzed in one Cox regression model simultaneously, both of these confounders proved to be statistically significant predictors of death-censored graft survival (Table 2). However, in such analysis, part of their impact is shared between the two confounders. The fact that information is present in both confounders also explains the drop in HRs when the model in which only one of these variables was considered was compared with the model in which both variables were considered (Table 2). Therefore, to answer the question whether PIRCHE-II or HLA mismatches add a significant benefit on top of the other, we investigated the effect of one score also within categories of the other score. The highest influence on outcome was observed in higher categories of the other variable. Consideration of the PIRCHE-II score improved the prediction of outcome significantly in patients with 4–6 HLA mismatches (Figure 3A), whereas HLA mismatches showed a significant influence in patients with a PIRCHE-II score >12 (Figure 4B). The grouping of patients with 4–6 and 0–3 HLA mismatches into two groups was justified because the HRs in the 4, 5, 6 MM groups differed from 1 by a large margin, whereas the HRs in the 0, 1, 2, and 3 MM subgroups were close to 1 (Table 3). Additional analysis of each HLA locus as a separate confounder did not lead to new insights toward our goal of comparing PIRCHE-II with HLA mismatches (Supplementary Table 3). Furthermore, we investigated whether the possibly more precise PIRCHE-II matching has potential to fully replace the currently used HLA A+B+DRB1 antigen matching. When analyzed simultaneously, both parameters remained statistically significant; however, HLA mismatches showed a higher prognostic value with a higher certainty than the PIRCHE-II score. Moreover, in the large subgroup of non-sensitized patients without alloreactive antibodies, HLA mismatches had a stronger impact on outcome than PIRCHE-II. These findings altogether indicated that the PIRCHE-II score cannot cover all immunological reasons for graft loss and that there are additional causes that can better be explained by HLA mismatches than by the PIRCHE score in its applied form. On the other hand, compared to the influence of HLA mismatches, the impact of PIRCHE-II on outcome was stronger in presensitized and pediatric patients, who are known to have a generally higher alloreactivity (21–25). Wan et al. reported a higher incidence of dnDSA at year 1 in patients with moderate (30%) and high immunological risk groups (29%) compared to the low-risk group (16%) (26). Lachmann et al. reported that a high PIRCHE-II score is a strong predictor of dnDSA development which is in agreement with our findings that the PIRCHE-II score is associated with an increased risk of graft loss in patients who have a higher alloreactivity and are thus more prone to dnDSA development (13).

As shown in Supplementary Figure 2, the Cox regression coefficients vary relatively broad around the best fit and indicate that, regarding its influence on outcome, there is room for improvement in the applied PIRCHE-II algorithm. Possible explanations for this observation are: (i) The current assumption that each additional epitope mismatch translates to the same amount of additional risk of graft loss may be too simplistic. (ii) The PIRCHE-II algorithm in this publication was based on only the HLA loci A, B and DRB1 that are currently used in kidney allocation. However, the PIRCHE-II score using low resolution typing results can also be calculated considering the HLA loci C and DQB1. Moreover, further improvement of PIRCHE-II-based matching is possible by expanding the PIRCHE-II algorithm to additional HLA loci typed at a higher resolution level. Theoretically, the algorithm could be extended to 11 HLA loci and these 11 loci could be typed two-field for every patient and donor. Furthermore, only DRB1 was utilized as the presenting HLA locus in this analysis and additional HLA class II loci, including HLA-DRB3/4/5, HLA-DQA1/HLA-DQB1, and HLA-DPA1/HLA-DPB1, could also be considered as the presenting loci, with potential impact on accuracy of prediction. However, in a large cohort, as analyzed by us, such an analysis would require an extremely high financial budget. Moreover, it must be considered that antigen matching may also benefit from high-resolution typing, which, however, has to be tested independently. Another possible issue might be the long time span of over 20 years. As shown in Supplementary Table 2, the PIRCHE-II effect was less pronounced in the earlier years compared to the newer period most probably due to less precise typing methods in the years from 1990 to 2003 that influence the amount of calculated epitope load much more compared to the amount of HLA mismatches.

The HLA A+B+DRB1 matching had a similar clinical impact as the PIRCHE-II score matching when HRs of both parameters were compared in the same model simultaneously (Table 2) or when, as shown in Figure 4 and in more detail in Supplementary Figure 3, their impact was evaluated in different categories of the other parameter. Overall, we found that the PIRCHE-II score could not outmatch the traditional HLA mismatch. However, our findings also indicate that if there are several possible recipients with the same HLA mismatch score, the PIRCHE-II score would be a decent tool to decide whom to give the organ; especially in the presence of a high HLA incompatibility with 4–6 mismatches (Figure 4A). This is especially important because recipients with 4–6 HLA mismatches made up as many as 37% of the analyzed collective and the intensifying admixture is expected to increase this number in the future. Therefore, every additional improvement resulting in better compatibility between the donor and recipient has the potential to improve the outcome further.

In conclusion, our results indicate that, especially in the presence of high grades of HLA incompatibility between the donor and recipient and in patients with high alloreactivity, PIRCHE-II matching which follows HLA matching is of additional value and can already be included into the kidney allocation algorithms. Whether a PIRCHE-II-based allocation which covers additional HLA loci typed at a higher resolution level can fully replace the traditional HLA matching must be addressed in a further, albeit costly study of a similarly sized large cohort.

## Data Availability Statement

The raw data are available upon request to the Collaborative Transplant Study in accordance with the consents of the patients and the participating transplant centers and registries.

## Ethics Statement

The work of the CTS is approved by the Ethics Committee of the Medical Faculty of Heidelberg University (No. 083/2005) and performed in accordance with the World Medical Association Declaration of Helsinki Ethical Principles in the currently valid version.

## Author Contributions

CS conceived and designed the study. MN provided the intersection to obtain the PIRCHE-II scores. CU analyzed the data and wrote the first draft of the manuscript. CS, BD, MN, and NL contributed to the revision of the manuscript. All authors approved the submitted version.

## Conflict of Interest

CS declares that he received a minor research grant from the PIRCHE AG. MN is an employee of PIRCHE AG that runs the PIRCHE web-portal. The remaining authors declare that the research was conducted in the absence of any commercial or financial relationships that could be construed as a potential conflict of interest.
