# Supplementary material for: Can PIRCHE-II Matching Outmatch Traditional HLA Matching?
Source: Front Immunol. 2021 Feb 26;12:631246. doi: 10.3389/fimmu.2021.631246 (PMC7952296; doi:10.3389/fimmu.2021.631246)

Supplementary Material

Supplemental Table 1. Demographics of 68,606 first deceased donor kidney transplantations according to the analyzed seven categories of PIRCHE-II score. For categorized variables *P*-values of chi-squared test and for continuous variables *P*-Values of Kruskal-Wallis test are shown.

Confounder	PIRCHE-II score category							*P*
	**0 – 1 n = 4,407**	**2 – 12 n = 5,223**	**13 – 25 n = 13,643**	**26 – 43 n = 19,996**	**44 – 68 n = 16,424**	**69 – 102 n = 7,511**	**> 102 n = 1,402**	
**Recipient sex**								<0.001
Male	2,691 (61.1%)	3,157 (60.4%)	8,456 (62.0%)	12,473 (62.4%)	10,577 (64.4%)	4,836 (64.4%)	943 (67.3%)	
Female	1,716 (38.9%)	2,066 (39.6%)	5,185 (38.0%)	7,513 (37.6%)	5,846 (35.6%)	2,675 (35.6%)	459 (32.7%)	
Unknown	0 (0.0%)	0 (0.0%)	2 (0.0%)	10 (0.1%)	1 (0.0%)	0 (0.0%)	0 (0.0%)	
**Recipient age (years)**								<0.001
Mean (SD)	48.4 (13.9)	47.8 (14.4)	48.1 (15.0)	48.9 (15.3)	49.9 (15.3)	50.9 (15.3)	53.4 (15.2)	
**Panel reactive antibodies (%)**								<0.001
0	2,815 (63.9%)	3,369 (64.5%)	8,395 (61.5%)	12,698 (63.5%)	10,699 (65.1%)	4,984 (66.4%)	927 (66.1%)	
1 – 20	302 (6.9%)	441 (8.4%)	1,200 (8.8%)	1,957 (9.8%)	1,656 (10.1%)	772 (10.3%)	169 (12.1%)	
21 – 99	179 (4.1%)	212 (4.1%)	463 (3.4%)	576 (2.9%)	485 (3.0%)	211 (2.8%)	32 (2.3%)	
Unknown	1,111 (25.2%)	1,201 (23.0%)	3,585 (26.3%)	4,765 (23.8%)	3,584 (21.8%)	1,544 (20.6%)	274 (19.5%)	
**Initial immunosuppression**								<0.001
Tac + MPA	1,161 (26.3%)	1,436 (27.5%)	4,289 (31.4%)	6,790 (34.0%)	5,556 (33.8%)	2,737 (36.4%)	569 (40.6%)	
CsA + MPA	1,228 (27.9%)	1,245 (23.8%)	3,074 (22.5%)	4,468 (22.3%)	3,768 (22.9%)	1,778 (23.7%)	310 (22.1%)	
Other	1,884 (42.8%)	2,306 (44.2%)	5,495 (40.3%)	7,579 (37.9%)	6,006 (36.6%)	2,494 (33.2%)	400 (28.5%)	
Unknown	134 (3.0%)	236 (4.5%)	785 (5.8%)	1,159 (5.8%)	1,094 (6.7%)	502 (6.7%)	123 (8.8%)	
**Initial induction therapy**								<0.001
ATG	294 (6.7%)	457 (8.7%)	1,245 (9.1%)	1,985 (9.9%)	1,802 (11.0%)	897 (11.9%)	170 (12.1%)	
IL-2RA	763 (17.3%)	968 (18.5%)	2,982 (21.9%)	5,129 (25.7%)	4,548 (27.7%)	2,251 (30.0%)	455 (32.5%)	
No Induction	3,147 (71.4%)	3,449 (66.0%)	8,369 (61.3%)	11,263 (56.3%)	8,557 (52.1%)	3,652 (48.6%)	609 (43.4%)	
Other	69 (1.6%)	113 (2.2%)	262 (1.9%)	460 (2.3%)	423 (2.6%)	209 (2.8%)	45 (3.2%)	
Unknown	134 (3.0%)	236 (4.5%)	785 (5.8%)	1,159 (5.8%)	1,094 (6.7%)	502 (6.7%)	123 (8.8%)	

Confounder	PIRCHE-II score category							*P*
	0 – 1<br>n = 4,407	2 – 12<br>n = 5,223	13 – 25<br>n = 13,643	26 – 43<br>n = 19,996	44 – 68<br>n = 16,424	69 – 102<br>n = 7,511	> 102<br>n = 1,402	
**Time on dialysis (months)**								<0.001
No Dialysis	92 (2.1%)	89 (1.7%)	250 (1.8%)	445 (2.2%)	368 (2.2%)	155 (2.1%)	44 (3.1%)	
Mean (SD)	41.3 (30.8)	44.4 (35.8)	45.8 (38.0)	45.3 (37.7)	45.3 (38.4)	45.7 (38.6)	43.1 (34.8)	
Unknown	1,124 (25.5%)	1,201 (23.0%)	3,385 (24.8%)	4,373 (21.9%)	3,146 (19.2%)	1,358 (18.1%)	216 (15.4%)	
**Donor sex**								0.16
Male	2,437 (55.3%)	2,919 (55.9%)	7,802 (57.2%)	11,430 (57.2%)	9,319 (56.7%)	4,257 (56.7%)	787 (56.1%)	
Female	1,962 (44.5%)	2,295 (43.9%)	5,824 (42.7%)	8,547 (42.7%)	7,092 (43.2%)	3,240 (43.1%)	614 (43.8%)	
Unknown	8 (0.2%)	9 (0.2%)	17 (0.1%)	19 (0.1%)	13 (0.1%)	14 (0.2%)	1 (0.1%)	
**Donor age (years)**								<0.001
Mean (SD)	45.0 (15.3)	45.4 (16.1)	46.4 (16.9)	47.4 (17.5)	48.3 (17.9)	49.1 (18.6)	52.5 (18.5)	
**Year of transplantation**								<0.001
1990 – 1996	776 (17.6%)	988 (18.9%)	2,029 (14.9%)	2,710 (13.6%)	2,037 (12.4%)	800 (10.7%)	107 (7.6%)	
1997 – 2003	1,466 (33.3%)	1,741 (33.3%)	4,225 (31.0%)	5,615 (28.1%)	4,533 (27.6%)	1,961 (26.1%)	346 (24.7%)	
2004 – 2010	1,346 (30.5%)	1,483 (28.4%)	4,045 (29.6%)	6,159 (30.8%)	5,076 (30.9%)	2,372 (31.6%)	458 (32.7%)	
2011 – 2016	819 (18.6%)	1,011 (19.4%)	3,344 (24.5%)	5,512 (27.6%)	4,778 (29.1%)	2,378 (31.7%)	491 (35.0%)	
**HLA A+B+DRB1 mismatches**								<0.001
0	4,074 (92.4%)	392 (7.5%)	3 (0.0%)	0 (0.0%)	0 (0.0%)	0 (0.0%)	0 (0.0%)	
1 – 2	332 (7.5%)	4,011 (76.8%)	6,935 (50.8%)	5,383 (26.9%)	1,789 (10.9%)	128 (1.7%)	1 (0.1%)	
3 – 4	1 (0.0%)	785 (15.0%)	6,098 (44.7%)	12,478 (62.4%)	11,559 (70.4%)	4,815 (64.1%)	531 (37.9%)	
5 – 6	0 (0.0%)	35 (0.7%)	607 (4.4%)	2,135 (10.7%)	3,076 (18.7%)	2,568 (34.2%)	870 (62.1%)	
**Cold ischemia time (hours)**								<0.001
Mean (SD)	17.8 (6.72)	18.0 (7.28)	17.5 (7.21)	17.0 (6.93)	16.8 (6.99)	16.4 (6.83)	15.8 (6.58)	

SD, standard deviation; Tac, tacrolimus; MPA, mycophenolic acid; CsA, cyclosporine A; ATG, anti-thymocyte globulin; IL-2RA, interleukin-2 receptor antagonist

Supplemental Table 2. The impact of adjusted PIRCHE-II scores and HLA mismatches on 5-year death-censored graft survival in subpopulations of kidney transplant recipients. Hazard ratios (HR), 95% confidence intervals (CI), and Wald statistic z value per adjusted PIRCHE-II score or per HLA A+B+DRB1 mismatch of two different multivariable Cox regression models with simultaneous or separate consideration of both parameters are shown.

**Subgroup**	**Simultaneous**				**Separate**			
	**HR**	**95% CI**	**Z**	***P***	**HR**	**95% CI**	**Z**	***P***
PRA=0 (n=43,887)								
Per adjusted PIRCHE-II score	1.047	1.012 – 1.084	2.6	0.008	1.118	1.090 – 1.146	8.8	<0.001
Per HLA A+B+DRB1 mismatch	1.082	1.051 – 1.113	5.4	<0.001	1.111	1.089 – 1.124	10.2	<0.001
PRA>0 (n=8,655)								
Per adjusted PIRCHE-II score	1.103	1.028 – 1.184	2.7	0.006	1.116	1.060 – 1.174	4.2	<0.001
Per HLA A+B+DRB1 mismatch	1.014	0.956 – 1.075	0.5	0.65	1.074	1.030 – 1.120	3.4	<0.001
Pediatric patients 0−17 years (n=2,671)								
Per adjusted PIRCHE-II score	1.092	1.012 – 1.086	1.3	0.18	1.114	1.008 – 1.231	2.1	0.034
Per HLA A+B+DRB1 mismatch	1.028	1.051 – 1.113	0.5	0.62	1.079	0.992 – 1.173	1.8	0.077
Adults ≥18 years (n=65,935)								
Per adjusted PIRCHE-II score	1.041	1.013 – 1.070	2.8	0.004	1.098	1.076 – 1.120	9.2	<0.001
Per HLA A+B+DRB1 mismatch	1.066	1.042 – 1.091	5.4	<0.001	1.092	1.074 – 1.110	10.5	<0.001
Cold ischemia time ≤18 hours (n=43,869)								
Per adjusted PIRCHE-II score	1.043	1.007 – 1.081	2.3	0.020	1.092	1.064 – 1.120	6.6	<0.001
Per HLA A+B+DRB1 mismatch	1.056	1.026 – 1.088	3.6	<0.001	1.082	1.060 – 1.105	7.3	<0.001
Cold ischemia time >18 hours (n=24,737)								
Per adjusted PIRCHE-II score	1.039	0.997 – 1.083	1.8	0.069	1.112	1.080 – 1.145	7.0	<0.001
Per HLA A+B+DRB1 mismatch	1.088	1.049 – 1.127	4.6	<0.001	1.114	1.080 – 1.145	8.4	<0.001
Donor age <60 years (n=50,443)								
Per adjusted PIRCHE-II score	1.044	1.011 – 1.078	2.6	0.009	1.098	1.073 – 1.123	8.0	<0.001
Per HLA A+B+DRB1 mismatch	1.066	1.036 – 1.097	4.4	<0.001	1.095	1.073 – 1.117	8.9	<0.001
*Donor age ≥60 years (n=*18,163*)*								
Per adjusted PIRCHE-II score	1.035	0.985 – 1.088	1.4	0.17	1.084	1.044 – 1.125	4.3	<0.001
Per HLA A+B+DRB1 mismatch	1.054	1.015 – 1.094	2.7	0.007	1.073	1.043 – 1.103	4.9	<0.001
Transplant year 1990–2003 (n=29,334)								
Per adjusted PIRCHE-II score	1.021	0.984 – 1.059	1.1	0.27	1.087	1.060 – 1.115	6.4	<0.001
Per HLA A+B+DRB1 mismatch	1.064	1.011 – 1.119	2.4	0.018	1.097	1.043 – 1.103	8.1	<0.001

Transplant year 2004–2016 (n=39,272)								
Per adjusted PIRCHE-II score	1.063	1.021 – 1.107	3.0	0.003	1.112	1.079 – 1.147	6.9	<0.001
Per HLA A+B+DRB1 mismatch	1.054	1.021 – 1.088	3.3	0.001	1.089	1.064 – 1.115	7.2	<0.001
Patients survival time >30 days (n=65,530)								
Per adjusted PIRCHE-II score	1.037	1.004 – 1.070	2.2	0.025	1.096	1.071 – 1.122	7.8	<0.001
Per HLA A+B+DRB1 mismatch	1.069	1.040– 1.098	4.9	<0.001	1.092	1.072 – 1.113	9.1	<0.001
Donor or recipient age <65								
Per adjusted PIRCHE-II score	1.041	1.012 – 1.070	2.8	0.006	1.094	1.073 – 1.116	8.8	<0.001
Per HLA A+B+DRB1 mismatch	1.065	1.039 – 1.091	5.0	<0.001	1.091	1.073 – 1.111	9.9	<0.001
Donor and recipient age ≥65								
Per adjusted PIRCHE-II score	1.039	0.938 – 1.152	0.7	0.46	1.084	0.996 – 1.180	1.9	0.062
Per HLA A+B+DRB1 mismatch	1.046	0.983 – 1.113	1.4	0.16	1.060	1.007 – 1.116	2.2	0.025

Supplemental Table 3. Impact of matching for PIRCHE-II score and HLA A+B+DRB1 mismatches on 5-year death-censored graft survival in different multivariable Cox regression models.

Model	HR	95% CI	Z	*P*
Without HLA A+B+DRB1 mismatches (AIC= 150,629)				
Per adjusted PIRCHE-II score	1.102	1.081 – 1.123	9.8	<0.001
Without adjusted PIRCHE-II score (AIC= 150,607)				
Per HLA-A mismatch	1.077	1.044 – 1.112	4.6	<0.001
Per HLA-B mismatch	1.094	1.057 – 1.132	5.2	<0.001
Per HLA-DR mismatch	1.118	1.082 – 1.156	6.6	<0.001
Simultaneously with both parameters (AIC= 150,598)				
Per adjusted PIRCHE-II score	1.046	1.018 – 1.075	3.2	0.001
Per HLA-A mismatch	1.046	1.001 – 1.085	2.4	0.015
Per HLA-B mismatch	1.062	1.022 – 1.104	3.1	0.002
Per HLA-DR mismatch	1.096	1.058 – 1.136	5.1	<0.001

HR, hazard ratio; CI, confidence interval; Z, Wald statistic value; AIC, Akaike Information Criterion

Supplemental Figure 1. Receiver operating characteristic curve analysis of 5-year graft loss for the full model. AUC, area under the curve.

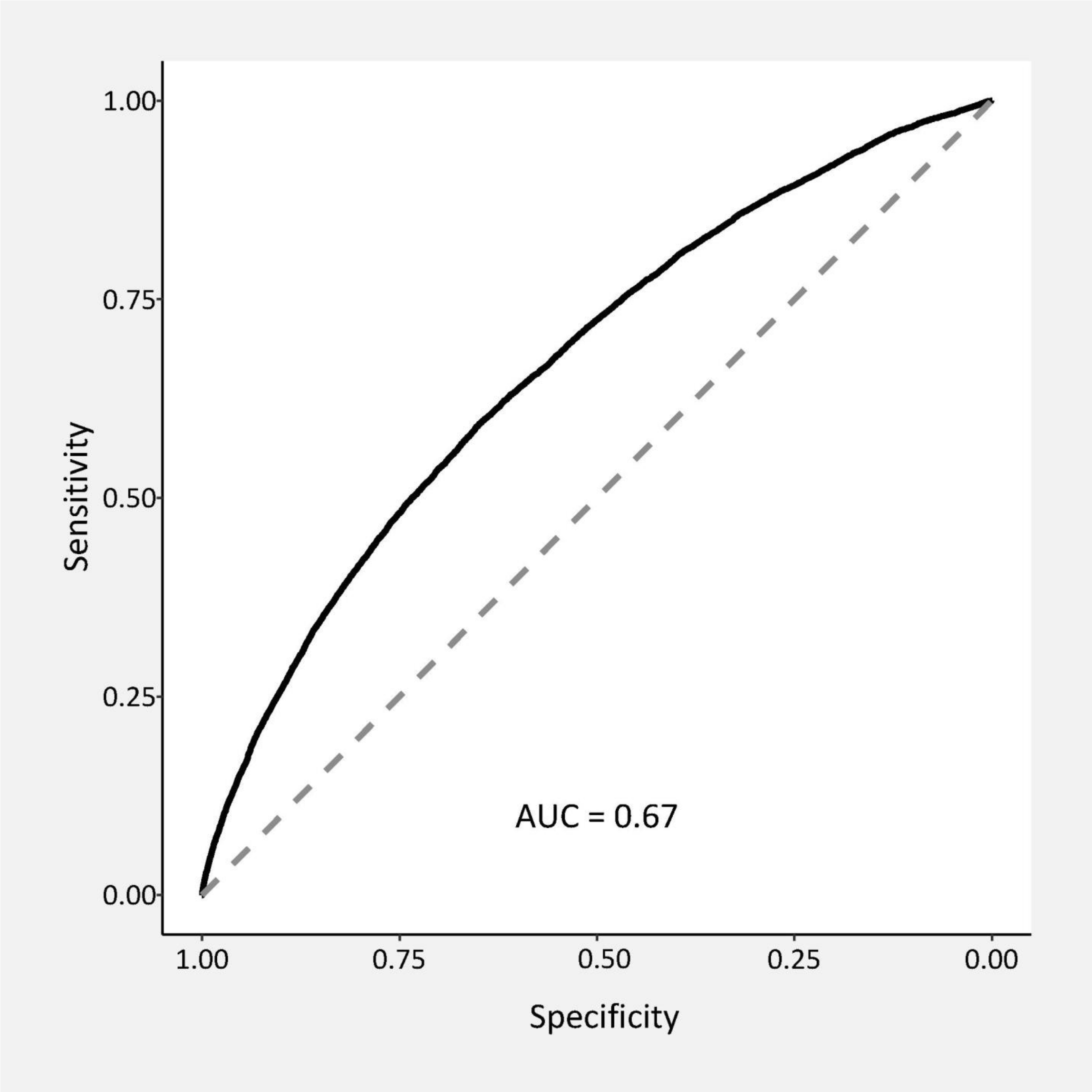

Supplemental Figure 2. The impact of PIRCHE-II score on 5-year death-censored graft survival in categories of kidney transplant recipients with a similar size of approximately 4,000 cases in reference to 0–1 PIRCHE-II score. Hazard ratios ± 95% confidence intervals are shown.

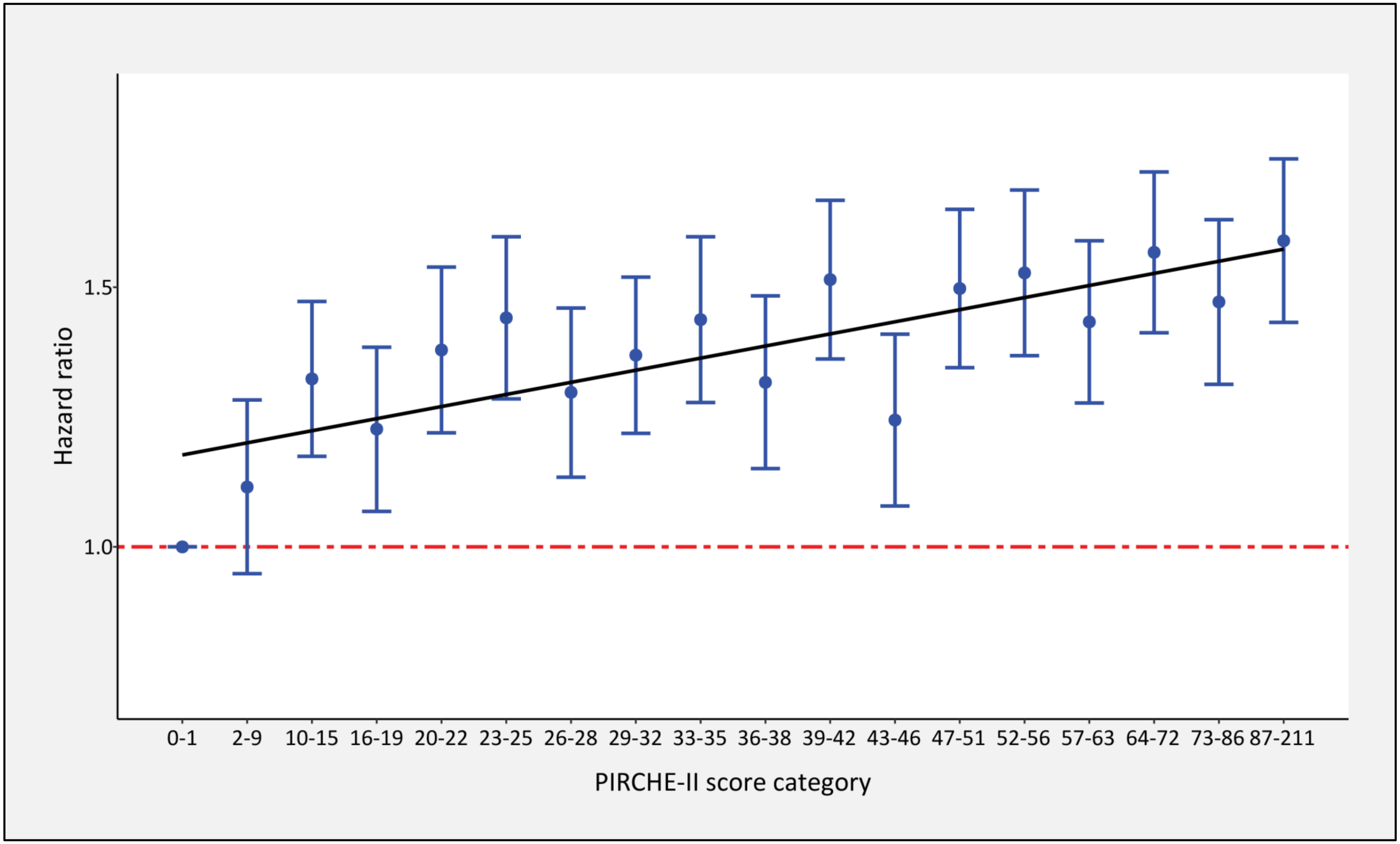

Supplemental Figure 3. The impact of adjusted PIRCHE-II scores (A) and HLA A+B+DRB1 mismatches (B) on 5-year death-censored graft survival. Results of Table 3 are illustrated graphically. Hazard ratios ± 95% confidence interval are shown.

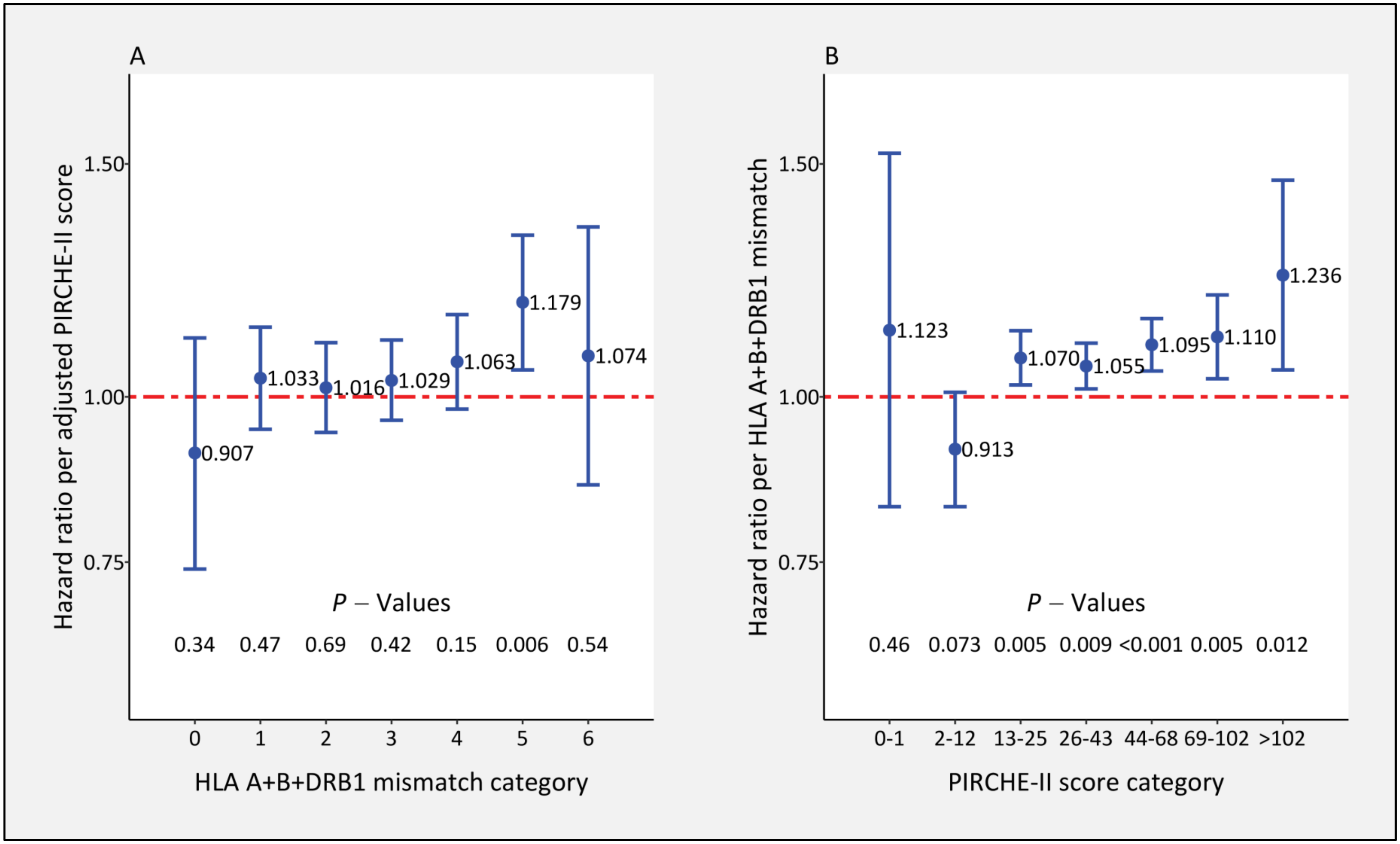

Supplement: Supplementary file 1 [file Table_1.pdf]
